# Knowledge, Attitude, and Practices (KAP) Regarding Meat Safety and Sanitation among Carcass Handlers Operating and Assessment of Bacteriological Quality of Meat Contact Surfaces at the Marrakech Slaughterhouse, Morocco

**DOI:** 10.1155/2022/4881494

**Published:** 2022-03-24

**Authors:** Mohammed Amine Bahir, Ikram Errachidi, Mouhssine Hemlali, Bouchaib Sarhane, Asmae Tantane, Alami Mohammed, Bouchra Belkadi, Abdelkarim Filali-Maltouf

**Affiliations:** ^1^Laboratory of Microbiology and Molecular Biology, Faculty of Sciences, Mohammed V University, Rabat, Morocco; ^2^National Institute of Hygiene, 27 Ibn Batouta street, BP 769 Agdal, Rabat, Morocco

## Abstract

According to the Moroccan Court of Auditors, the meats are prepared in slaughterhouses that do not meet the basic conditions required by Moroccan standards. This survey is being conducted to assess the knowledge, attitudes, and practices of handlers regarding the salubrity and hygiene of meat and to evaluate the bacteriological load of work surfaces in a slaughterhouse located in the Marrakech region. A total of 100 people working at the slaughterhouse participated in the study. The average values concerning the attitude and practice of the carcass handlers were, respectively, very satisfactory (65.7%) and acceptable (53.44%), while the average value of knowledge was generally low (39%). Bacterial load was assessed by the serial dilution method using the standard procedure. Seventy samples were taken from the hands of manipulators, knives, clothes, hooks, door handles, floor, and walls over an area of between 20 and 100 cm^2^. The total number of aerobic mesophiles (TAVCs), *Escherichia coli*, *Staphylococcus aureus*, *Pseudomonas aeruginosa,* and *Salmonella* spp was determined for each sample. *Escherichia coli* was the predominant isolate (42%), while *Salmonella* spp and *Pseudomonas aeruginosa* were the least bacterial isolates with 16% and 14%, respectively. Walls and knives were the most contaminated by E. coli at 90%. This survey reveals the importance of developing formal training for all slaughterhouse handlers regarding meat hygiene and safety during carcass processing to develop their knowledge and practices. Bacteriological results indicate a need to improve the available slaughter facilities and develop an appropriate slaughter process strategy to minimize the risk of carcass contamination.

## 1. Introduction

Foodborne illnesses have caused significant morbidity and mortality worldwide, particularly in developing countries, and are a considerable obstacle to socioeconomic development worldwide [1]. The first global estimates of foodborne illness released to date show that 1 in 10 people get sick from eating contaminated food every year, and 420,000 die from it [2]. Food safety is a matter of great concern and importance to public health, especially when food is handled in a highly contaminated environment [3]. Many factors such as lack of hygiene and financial resources to invest in safer equipment, poor food handling practices, as well as lack of education of handlers in food establishments such as slaughterhouses contribute significantly to the increased incidence of foodborne illness [4, 5]. Meat handlers such as butchers working in slaughterhouses can not only be the main vectors of meat contamination but can also be an asymptomatic reservoir of foodborne microorganisms [6, 7]. The possible sources of contamination with these microorganisms are the animal's skin, the surfaces in contact with meat, the clothing, and the hands of personnel involved in the slaughter process. Gram-negative bacteria have been reported to account for around 69% of cases of foodborne bacterial illness [8]. Coliforms are the most frequently identified group on meat, especially *Citrobacter freundii* and *Escherichia coli*, while other microorganisms are less frequent such as *Klebsiella*, *Salmonella*, *Shigella sonnei*, *Proteus* spp and *Staphylococcus aureus* [9, 10]. These microorganisms can be transferred to food during processing, packaging, preparation, and serving by touching, breathing, coughing, or sneezing [11, 12]. Accordingly, every food handler should maintain a good level of personal hygiene and cleanliness and excellent hygiene practices to ensure that cross-contamination can be reduced [5]. In Morocco, an average of 100 Foodborne Disease Outbreak (FBDO) episodes is reported annually, corresponding to 1500 cases notified in all provinces and regions of the kingdom. However, the laboratory confirmation rate remains very low, not exceeding the 10% threshold. According to the Department of Epidemiology and Disease Control (DEDC), during 2007 to 2017, 13778 cases of FBDO were identified, of which 57.1% of the households were declared in a family environment, and 42.9% of these outbreaks were reported in communities [13]. In Morocco, very little data is published on the situation of manipulators working in the slaughterhouses environments, as well as their knowledge, attitudes, and practices. The objective of this study is to provide knowledge on the effect of hygiene on the bacteriological load of surfaces in contact with meat in order to raise awareness for the responsible authorities to develop strategies to improve food safety in national slaughterhouses.

## 2. Materials and Methods

### 2.1. Study Area

This study constitutes the first work carried out at the national level to shed light on the knowledge, attitudes, and practices in terms of hygiene and handling of meat among handlers in a municipal slaughterhouse located in the region of Marrakech. The studied slaughterhouse is located in the region of Marrakech in Morocco, which was created during French colonization. It is located inside the urban agglomeration of Douar el Askar and occupies approximately 2000 m^2^. It includes a stall area, two cattle slaughter rooms, a room for sheep, a cold room, and administrative offices. The administrative management of these slaughterhouses is carried out by the Ministry of the Interior, while veterinary inspection is carried out by the National Office for Food Safety (ONSSA). The slaughterhouse operates all the week except Friday. Slaughtering usually takes place in the morning between 03 and 12 a.m. The number of people working in the slaughterhouse is variable. The butchers coming from outside to slaughter their own animals pay other temporary workers to handle these carcasses. In 2019, a total of 30,000 cattle and 66,000 sheep were slaughtered.

### 2.2. Survey

The survey was carried out between January and May 2019. A total of 100 randomly selected employees working in cattle or sheep slaughterhouses were assessed. The workers participating in the survey were randomly selected according to their wishes. No one was forced to participate in the investigation. The distribution of the participants is presented in Table 1: 54 (54%) work in the slaughter of sheep, and 46 (46%) slaughter cattle. No participants reported mixed culling. The hygiene knowledge, attitude, and practice levels of handlers were determined by face-to-face interviews and through direct observation of handler hygiene status and practices. The study was supplemented by bacteriological analysis using sterile swabs taken from meat contact surfaces to count colonies with the intention of colony count and identify pathogenic bacteria.

### 2.3. Questionnaire

A structured questionnaire with four parts was developed based on previous research to assess the sociodemographic characteristics, knowledge, attitudes, and slaughter practices of handlers in meat safety and sanitation procedures [14, 15]. All participants were interviewed face to face to ensure the accuracy of the responses. The questions were read aloud during the interview. Respondents were given sufficient time to answer each question.

The information on the sociodemographic characteristics of the workers mainly concerned the slaughterhouse working area, age, level of education, duration of employment, participation in hygiene training, and their health status. The information on workers' knowledge of food safety includes 10 questions on the risks of microbiological contamination of carcasses, the importance of refrigeration and personal hygiene, and the risks associated with foodborne illnesses. The participants had the choice to answer “true” or “false” and “not sure”. The section on the attitudes of manipulators also includes ten questions that respondents could answer “agree” or “not sure”. The slaughter practice section had 18 questions on good slaughter practices, the wearing of personal protective equipment, and personal hygiene during slaughter operations. Respondents were invited to respond by “yes” or “no”.

### 2.4. Sample Collection

A set of 70 samples were randomly taken aseptically from the hands of manipulators, ground, wall, door wrist, knives, clothes, and hooks. 10 different samples of each point were taken according to the ISO 18593:2018 standard. An area of 20 cm^2^ was taken from the hooks, hands, knives, and door of the different doors, such as the toilet doors, the locker room door, and the door of the slaughterhouse, while 100 cm^2^ was taken from the walls, grounds, and clothing of the slaughterers by swabbing. The swab is placed in a sterile capped tube containing 10 ml of normal saline; then, the rod is broken under aseptic conditions. Each swab is numbered appropriately, indicating site and date of collection. The samples taken were transported directly to the Laboratory of Microbiology and Molecular Biology located at the Faculty of Sciences of Rabat for microbiological analysis, following biosafety and biosecurity instructions. The swab is placed in capped sterile tube containing 10 ml of normal saline solution, and then, the rod is broken up under aseptic conditions. Each swab is numbered appropriately, indicating the site and date of collection. The samples taken were transported directly to Laboratory of Microbiology and molecular Biology localized in the Faculty of Sciences in Rabat for microbiological analyses, following the biosafety and biosecurity instructions.

### 2.5. Identification of Bacteria

Once in the laboratory, the swabs are vortexed for 30 seconds to ensure mixing of the sample. Serial dilutions were made in sterile 0.1% peptone water. Total mesophilic aerobic count (TAVC), *Escherichia coli*, *Staphylococcus aureus*, *Pseudomonas aeruginosa*, and *Salmonella* spp were carried out according to the methods specified in the FDA [16]). Colony-forming units (CFU) per cm^2^ of the sample were calculated using each's dilution factor and converted to log10CFU/cm^2^ values. The mean values of the total viable aerobic counts in log10CFU/cm^2^ were determined and reported as averages.

### 2.6. Data Management and Analysis

The data collected from thestudy area and the results of the laboratory investigations were entered intoXLSTAT and analyzed[KC1] . The statisticalsignificance for all tests was set at the level of p ≤ 0.05 using descriptivestatistics.

## 3. Results

### 3.1. Questionnaire Survey

#### 3.1.1. Sociodemographic Profile of Carcass Handlers Operating

All of 100 food workers which participated in this study were male. The majority of them were young people between 18 and 30 years old (42%). About 37% of them had below high school education, 23% was illiterate, 22% had illiterate, 15% had secondary education, and 3% had higher education but not necessarily related to food. The duration of employment was variable. The majority had worked for more than 5 years. All manipulators in our study (100%) did not take training regarding meat hygiene and handling practices. The majority (84%) wish to undertake training (Tables 1 and 2).

#### 3.1.2. Medical Situation of the Workers

Regarding the medical situation of the handlers in this survey, the majority (93.75%) did not have a medical certificate, and 84% have never had a health check by the administration of the slaughterhouse. Only those who have taken a preemployment test (16%) continue with medical testing. However, just 10% of manipulators perform medical checks every three months, 17% every six months, 31% every 12 months, 34% if necessary, while 8% have never had a check-up before (Table 3).

#### 3.1.3. Handler's Hygiene Knowledge

The food safety knowledge of food handlers was poor. The mean food safety knowledge scores was 39%. This disagrees with [5] in Cameroon who found an average of 49.02% and [17] with high level of knowledge in India. In this study, although 54%, 58%, and 65% of the respondents had correct answers to questions about risks that can be caused by microorganisms and by manipulators who have a diarrheal syndrome, most of the respondents (64%) failed to select the correct answer about the contamination of carcasses by direct contact with work surfaces. However, all responses about the effect of microorganisms on health (40%) and the risk of contamination generated by water (11%) and hoses used during cleaning slaughtering operations (8%) were generally poor. In relation with the conservation of meat, only 30% of workers had correct answers (Table 4). A study by [18] found that food handlers did not take into temperature control during storing food. According to these results, we can conclude that these workers have a lack of food safety knowledge which can lead to an increase in the number of contaminations. For these reasons, workers should enter an educational program related to food safety and take courses related to their work position. Also, these workers should have an evaluation after finishing the program.

The results obtained are very positive, where the average value of the responses found in our study is 65,7%, in agreement with almost all previous studies [18, 19]. Most of the questions asked were correct. The handlers are aware of the obligation to wash hands after using the toilet (80%). This agrees with the results of [1] where 100% of handlers declared that it is important to wash hands before slaughter operations. Most manipulators believe in the vital role of training (84%) and disinfection of slaughterhouses to avoid contamination of carcasses (87%). These percentages were higher than the on reported by [19]. A majority of 60% of manipulators agree with the importance of wearing protective equipment, 87% and 76% of them, respectively, are aware of the importance of washing their seats before and after slaughter operations.  Table 5 shows that 25% of workers use their hands with injury and without any precaution of safety; only 6% of the workers are aware of the need for an evaluation to ensure effectiveness.

The average value of the answers obtained related to carcass handlers' practices was 53,44%. This is similar to the results conducted by [5] unlike the result shown by [17]. The present study revealed that 100% of the workers did wash their hands before working, and no one declared that they use disinfectant. Only 43% of manipulators declared washing their hands with soaps after using the restroom. This is well supported by the reports of [4]. The lack of disinfection of equipments at the workplace will increase the risk of dissemination of pathogenic agents and promote cross-contamination of carcasses by handlers as all slaughter operations are done only by handlers in one place in the ground. When handling carcasses, 25% and 32% of manipulators declared, respectively, that they work with injuries on their hands and sick or suffering from diarrhoeal syndrome. In the same way, [20] declared that 45,6% of respondents continued working despite having foodborne illnesses. The survey shows the lack of knowledge of the workers concerning contamination on the carcasses. Meat handlers are like butchers working at the slaughterhouse may not only constitute the main vehicle of meat contamination but may also be an asymptomatic reservoir of foodborne microorganisms [5]. In addition, most respondents keep their nails long (90%) that helps them during skin removal without knowing that it could lead to be a reservoir for microorganisms. Almost all food workers were unaware of the critical role of general sanitary practices in their work places, such as using gloves (100% wrong answers), using aprons (95% wrong answers), the contact of carcasses with the slaughter environment (95% wrong answers), and daily cleaning of slaughter equipment (89% wrong answers). This disagrees with the work obtained by [21] in Brazil, who announced that out of 166 food handlers who participated in the interviews, 66.3% declared using an apron and 70,5% used gloves during slaughter operations. Most of the workers in the slaughterhouse announced that it was not practical to use gloves during slaughter operations. These unhygienic practices such as wearing dirty clothes, not using gloves, deporting carcasses into the ground during slaughter operations could lead to cross-contamination by pathogenic microbes making the meat dangerous for the consumer [22] (Table 6).

#### 3.1.4. Standard Plate Count and Bacterial Isolation

Meat is the most perishable of all important food since it contain sufficient nutrient needed to support the growth of microorganisms [23]. The results of this study showed that that mean standard total plate count obtained from all points analyzed were similar. The highest log mean value of TVC (Total viable count) was observed on the wall (4.43 log10 CFU/cm^2^), and the lowest mean values were observed on the hook (2.18 log10CFU/cm^2^) (Table 7). The averages obtained were low compared to other works obtained by ([22, 24]. *Escherichia coli* was the predominant isolate (42%) followed by *Staphylococcus aureus* (28%). The least bacterial isolates were *Salmonella* species with 14% (Table 8). The bacterial contaminants of meat samples in the study were *E. coli*, *Staphylococcus aureus, Pseudomonas aureuginosa* and *Salmonella* spp. Similar bacterial contaminants have been reported by [4]. The higher rate of contamination of meat with these organisms is an indication of deplorable state of poor hygienic and sanitary practices employed right from the slaughtering, transportation to butcher shops, and processing.

The high microbial load obtained from wall is an indication of the ineffectiveness of the method used in cleaning, which are usually washed with water only. Moreover, during the slaughtering process, the handlers rest their hands on wall and touch them with different slaughtering instruments. This is consistent with the respondent's statement obtained in the survey, or most of them (95%) declared having touched the walls during their movements within the slaughter rooms.

During the sampling of this study, the grounds were completely soaked with blood. In addition, all the stages of slaughter are done in the same place. Moreover, the movements of the manipulators between the clean and soiled sectors could explain the highest results obtained for isolation of *Pseudomonas aeruginosa (*80%) and *Salmonella* spp (50%) (Table 8).

The number obtained from butcher's knives in this study is almost low to values of obtained by [25] who reported total viable count of 6.16 log10CFU/cm^2^. The higher levels of TVC and *Escherichia coli* in handling equipments is an indication of inadequate cleaning and poor disinfection. The high microbial load on the knife is an indication of inadequate cleaning and poor or absence of sterilization, continuous use of a single knife despite contact with dirty or contaminated surfaces, and lack of separation between clean and dirty processes. This has been confirmed during the survey, or 31% of manipulators declared that they deposit their knives on the ground. The presence of bacterial pathogens in meat contact surfaces may contribute to the contamination of meat [26].

Protective cloth is important in the butcher shops to reduce the chance of contamination. In order to protect both food products and meat handlers from cross contamination, the manipulators should wear protective clothes while working. In this study, almost of manipulators did not wear their apron (95%). This may explained the presence of bacterial pathogens in their cloth.

Although 100% of respondents reported washing their hands with water, the total mean bacterial load obtained was 3.57 ± 0.01 CFU/cm^2^. This result could be explained by the quick contamination of their hands once they begin the slaughter, and they did not wear gloves for this act. These results were lower than the value reported by [22] which was 5.83 CFU/cm^2^ obtained from worker's hands after processing of the meat from various butcher shops located in 6 markets in India. This may be confirm the lack of knowledge obtained on the survey and explain the direct impact on the bacterial load.

The high microbial load on the processing facility surfaces in this study underscores the poor level of personnel hygiene and poor sanitation at the slaughterhouse. The personnel working in Marrakech slaughterhouse did not apply hygienic practices which is mainly due to lack of knowledge. Based on the bacteria isolated and bacterial load on different surfaces in the slaughterhouse, meat could be contaminated by contact with contaminated surfaces and equipments in the slaughterhouse to pose public health hazards. Thus, to safeguard the public against the risks of foodborne bacterial infections, there is a need to educate and advocate for practicing good sanitation and meat handling techniques for all the manipulators.

## 4. Conclusions

In conclusion, this study contributes to showcasing the level of knowledge, attitudes, and practices of manipulators as well as the current situation of a slaughterhouse located in the region of Marrakech in Morocco. The low level of knowledge and the bad practices applied by the handlers and the high loads of bacteria isolated from most of the surfaces studied show that the meat could easily become contaminated during the slaughter process. The establishment of training on hygiene and good slaughtering practices, slaughter facilities, and cleaning processes are the most essential points to report in this study.

## Figures and Tables

**Table 1:** Distribution of sociodemographic characteristics of the respondents.

Variables	% (*n*)
*Slaughter area*	
Sheep	54 (54)
Cattle	46 (46)

*Age*	
<18 yo	2 (2)
18 -30 yo	42 (42)
31-40 yo	26 (26)
41-60 yo	27 (27)
>60 yo	3 (3)

*Education level*	
Illiterate	23 (23)
Primary education	22 (22)
Secondary education	15 (15)
High school	3 (3)
Academic	37 (37)

*Working period*	
0-4 years	12 (12)
5-10 years	29 (29)
11-15 years	14 (14)
15-20 years	13 (13)
>20 years	32 (32)

**Table 2:** Handler training in meat hygiene and handling.

Variables	% (*n*)
*Hygiene training of handlers*	
Yes	0 (0)
No	100 (100)

*Worker's appreciation of the willingness to follow hygiene-training*	
Yes	84 (84)
No	16 (16)

**Table 3:** Medical situation of the workers.

Variables	% (*n*)
*Possession of medical certificate*	
Yes	6,25 (5)
No	93,75 (75)

*Preemployment medical chech-up by administration for workers*	
Yes	16 (16)
No	84 (84)

*Respondents who followed medical checks in the slaughterhouse*	
Yes	16 (100)
No	0 (0)

*The last health check carried out*	
1 month	15 (15)
3 months	23 (23)
6 months	13 (13)
12 months	20 (20)
>12 months	21 (21)
Never	8 (8)

*Health check interval*	
Every 3 months	10 (10)
Every 6 months	17 (17)
Every 12 months	31 (31)
If necessary	34 (34)
Never checked before	8 (8)

**Table 4:** Carcass handlers' knowledge on food hygiene and sanitation.

The statements	Correct answers % (*n*)
Can meat spoilage be caused by microorganisms?	54
Is the contamination of meat very risky due to the shelf life?	58
Could unsanitary practices be a source of carcass contamination?	40
Can contamination be caused by direct contact between bare hands and animals or materials?	36
Does the chilling of meat at temperatures below 20°C contribute to delaying microbial deterioration?	30
Can microbial contamination cause serious illness leading to hospitalisation and sometimes death?	40
Can healthy carriers carry microbes?	48
Can a handler with diarrhoeal syndrome be a source of risk?	65
Can water be a source of microbial contamination?	11
Can water from hoses used for cleaning be a source of contamination of carcasses?	8
Average knowledge estimate∗	39%

**Table 5:** Carcass handlers' attitude on food hygiene and sanitation.

The statements	Correct answer, % (*n*)
Hand washing after the toilet with a disinfectant is mandatory	80(80)
The handler must check his state of health	88 (88)
Handling meat with lesions on the hand is a risk of contamination	25 (25)
	6 (6)
Training is a very interesting for me	84 (84)
Disinfecting abattoir premises is a way to avoid contamination	87 (87)
The wearing of protective equipment (apron) is necessary	60 (60)
Cleaning the slaughter area before slaughter operations	87 (87)
Cleaning of equipment before slaughter is desirable	76 (76)
The deposit of organ meats on the ground is prohibited	64 (64)
Average attitudes estimate∗	65,7%

**Table 6:** Carcass handlers' practices in food hygiene and sanitation.

Statements	Answers, % (*n*)	
Variables	Correct	Wrong
Hand washing before handling	100(100)	0(0)
If so, do you wash them with disinfectant?	0(0)	100(100)
Do you wash your hands every time you use the restroom?	98(98)	2(2)
With or without soap?	43(43)	57(57)
Do you handle carcasses when you have injuries to your hands?	25(25)	75(75)
Do you handle carcasses when you are sick or suffering from diarrhoeal syndrome?	32(32)	68(68)
Do you keep your finger nails long?	90(0)	10(10)
Do you wear gloves during slaughter?	0(0)	100(100)
During slaughter, is there contact with the skin, walls, floor, or equipment?	5(5)	95(95)
Do you use an apron during the process?	5(5)	95(95
Do you use boots during slaughter?	69(69)	31(31
Do you place the cutters and winch on the floor?	69(69)	31(31)
Are carcasses and offal placed in direct contact with floors, walls, or other equipment during hide removal and transport operations?	64(64)	36(36)
Do you clean slaughter equipment daily?	11(11)	89(89)
Cleaning of the area before	87(87)	13(13)
The cleaning of the area after	97(97)	3(3)
Cleaning the front knives	76(76)	24(24)
Cleaning the knives after	91(91)	9 (9)

**Table 7:** Standard plate count from swabs of slaughter surface after slaughter process.

Sample type	Number of sample	Mean count log10CFU/cm^2^	Minimum count log10CFU/cm^2^	Maximum count log10CFU/cm^2^
Ground	10	3.65	2.90	3.96
Wall	10	3.84	2.86	4.43
Door wrist	10	3.62	2.81	4
Knives	10	3.46	2.30	3.78
Hands	10	3.57	2.62	4
Clothes	10	3.45	2.20	3.88
Hook	10	3.61	2.18	4

**Table 8:** Isolated bacteria from swabs of slaughter surface after slaughter process.

Isolated bacteria	Ground	Wall	Door wrist	Knives	Hands	Clothes	Hook	Total
*Escherichia coli*	60%	90%	30%	90%	80%	50%	60%	42% (46)
*Staphylococcus aureus*	40%	50%	30%	40%	60%	40%	50%	28% (31)
*Pseudomonas aeruginosa*	80%	10%	10%	10%	10%	0%	20%	13% (14)
*Salmonella species*	50%	0%	0%	30%	30%	40%	10%	14% (16)

## Data Availability

The authors declare that they have all the necessary data and are available where appropriate or requested by the editor.
